# miR-125a-5p-abundant exosomes derived from mesenchymal stem cells suppress chondrocyte degeneration via targeting E2F2 in traumatic osteoarthritis

**DOI:** 10.1080/21655979.2021.1995580

**Published:** 2021-12-02

**Authors:** Qingqing Xia, Quan Wang, Feng Lin, Junjuan Wang

**Affiliations:** a Department of Laboratory Medicine, Huangyan Hospital of Wenzhou Medical University, Taizhou First People’s Hospital, Taizhou, Zhejiang Province, China; b Department of Orthopaedics, The Second Affiliated Hospital and Yuying Children’s Hospital of Wenzhou Medical University, China; c School of Basic Medical Sciences and Forensic Medicine, Hangzhou Medical College, Hangzhou, Zhejiang, China; d Department of General Surgery, Huangyan Hospital of Wenzhou Medical University, Taizhou First People's Hospital, Taizhou, Zhejiang, China

**Keywords:** Traumatic osteoarthritis, chondrocyte degeneration, bone marrow mesenchymal stem cells, exosomes, miR-125a-5p, E2f2

## Abstract

miRNAs are broad participants in vertebrate biological processes, and they are also the major players in pathological processes. miR-125a-5p was recently found a modulator in the progression of osteoarthritis (OA). Our study was aimed to explore the role and underlying mechanisms of miR-125a-5p-abundant exosomes derived from mesenchymal stem cells (MSC) on OA progression. We separated bone marrow mesenchymal stem cells (BMSCs) as well as the exosomes from traumatic OA patients. The immunofluorescence and cartilage staining were implemented for the observation and the assessment on endocytosis of chondrocytes and exosomal miR-125a-5p efficacy to cartilage degradation. Dual luciferase reporter assay was performed to verified the relationship between miR-125a-5p and E2F2. Then, the function of exosomal miR-125a-5p were examined on chondrocyte degeneration *in vitro* and *in vivo*. Our findings indicated that E2F2 expression was elevated while the miR-125a-5p was down in traumatic OA cartilage tissue, showing a negative correlation of the former and the latter. miR-125a-5p targets E2F2 in traumatic OA cartilage tissue and leads to the down-expression of E2F2. The E2F2 expression in chondrocytes was decreased after internalization of exosomes. We additionally found that BMSCs-derived exosomes were rich in miR-125a-5p content and chondrocytes can have it internalized. miR-125a-5p is endowed with a trait of accelerating chondrocytes migration, which is going along with the up-expressions of Collagen II, aggrecan and SOX9 and the down-expression of MMP-13 *in vitro*. Besides that, the mice model with post-traumatic OA turned out that exosomal miR-125a-5p might beget an alleviation in chondrocyte extracellular matrix degradation. All these outcomes revealed that BMSCs-derived exosomal miR-125a-5p is a positive regulator for chondrocyte migration and inhibit cartilage degeneration We thus were reasonable to believe that transferring of exosomal miR-125a-5p is a prospective strategy for OA treatment.

## Graphical abstract

**Figure:**
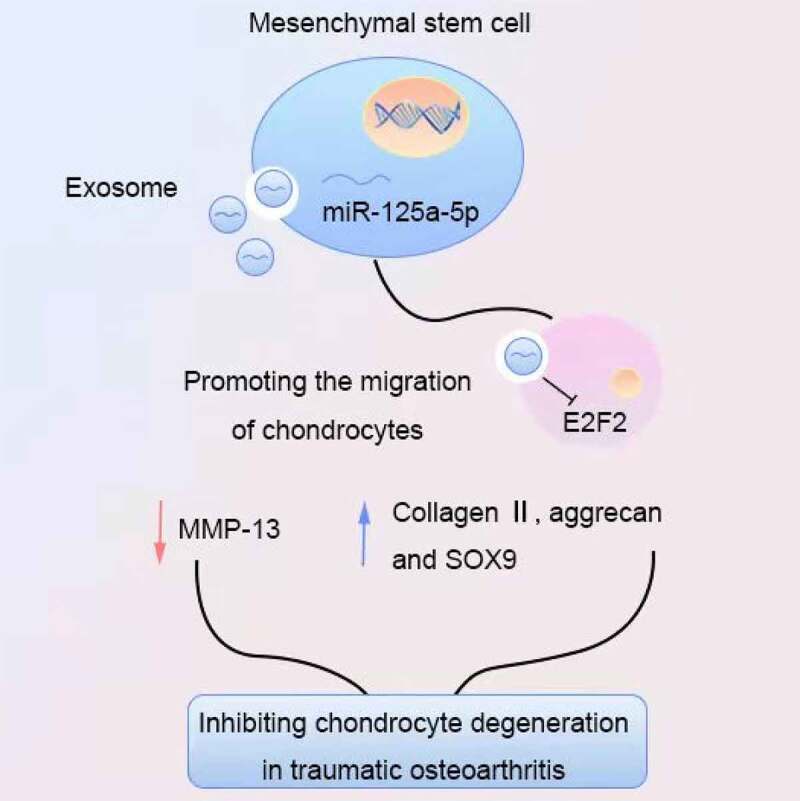

## Background

Osteoarthritis (OA), the prevalent joint disease usually happens to aging population has brought a huge burden to numerous families as well as society [1]. OA is mainly resulted from the gradual runoff in chondrocytes, which are the sole material in organismal articular cartilage supplement [2]. Clinical explanation to OA occurrence as well as its development currently is still vacant [2], though there have been guidelines provided for the decision-making of clinicians and patients in OA treatment; last but not the least, the joint replacement surgery for OA remains far from clinical practice [3]. The extracellular vesicles that are released from cells with their diameters of 30–100 nm are the ones defined as exosomes [4]. Exosomes mainly function as the intercellular communicators within cells and cells, and they transport the biologically active molecules from one to another [4]. It was found in mesenchymal stem cells (MSC) that their exosomes would relieve the pathological development of OA [5]. Such as the exosomes generated by embryonic stem cells-MSCs are the slow-release factors in OA development by a way of holding the balance amid chondrocyte extracellular matrix synthesis and the degradation [6]. In addition, cartilage contains no blood vessels, leaving it with little ability to repair itself, while exosomes have been shown to promote chondrocyte migration and thus enhance regeneration of damaged tissues [7]. MSC-derived exosomes are also able to help repair damaged joints, and thus they are deem as the interrupter in joint diseases development [8]. Hence, a treatment to OA with the administration of MSC-derived exosomes conduces to repairing in temporomandibular joint and accelerating the regeneration [8,9]. More and more studies have shown that microRNA is a potential biomarker for various diseases and plays an important role in regulating the development of OA [9–11].

For example, miR-29b-3p interplays with PGRN by targeting its 3ʹUTR so as to facilitate the apoptosis of chondrocyte in OA [11]; miR-365, the one regarded as an effective therapeutic molecule, is targeted to be turned down in both the prevention from and the treatment to osteoarthritis [12]; furthermore, development of OA could be also suppressed by intra-articular administration of antago-mir-483-5p, where the expressions of ECM enzymes Matrilin3 and metalloproteinase 2 are manipulated [13]. miR-125a-5p is recently found associated with OA, which is silenced to down-regulate both the expressions of inflammatory factors and chemokines [14]. It was predicted by an online software that gene E2F2 is a possible target of miR-125a-5p [15]. E2F2 is an important member of the E2F transcription factor family, which takes part in the process of cell proliferation, differentiation and apoptosis [16]. E2F2 is highly expressed in osteoarthritis synovial tissue [17]. The E2F2 gene is located at 1p36, and the relative molecular mass of the encoded protein is 47.5kDa [16]. E2F2 has a pocket protein (pRB) binding domain to which pRB binds [18]. At the end of the cell cycle G1, the phosphorylation of pRB caused by cyclins (CDKs) will decompose the pRB/E2F2 complex, thereby releasing and activating E2F2, regulating downstream gene expression (such as encoding proteins and enzymes required for DNA replication), and ultimately affecting Cell viability [18]. E2F2 is a regulator in cellular proliferation, differentiation, apoptosis, and cytokine synthesis and secretion in osteosarcoma cells and gliomas, and ultimately affects the pathological process of tumors [19,20]. Gene E2F2 has been proved the core player in OA cartilage degradation after the trauma in vivo.

Contextually, we assumed that mesenchymal stem cell-derived exosomal miR-125a-5p may be target E2F2 to ameliorate OA. Hence, the present study aims to investigate the regulatory mechanism of MSC-derived exosomal miR-125a-5p inhibiting chondrocyte degeneration of traumatic OA by targeting E2F2. We attempted to provide a new insight to the clinical study as well as the treatment of OA.

## Methods

### Ethics statement

Our study has been authorized by the ethics committee of Hangzhou medical college and The second affiliated hospital and yuying children's hospital of  Wenzhou medical university. This study complies with the Declaration of Helsinki, and all patients signed the informed consent. Animal usage protocol in this study was approved by the animal ethics committee. Databases of TargetScan (http://www.targetscan.org/vert_71/) and StarBase (http://starbase.sysu.edu.cn/) were our primary methods in the predictions to target genes and corresponding regulation pathways. Patients were enrolled in the group from June 2017 to June 2019.

A total of 30 cases of cartilage tissue of knee traumatic OA patients and 30 cases of traumatictraumatic amputation patients’ knee cartilage tissue were collected. Patients with rheumatoid arthritis and septic arthritis were excluded. This research. Thirty patients with traumatic osteoarthritis were enrolled, including 12 males and 18 females, with an average age of 56.55 years old and 47–69 years old. There were 30 cases of traumatic amputation, including 17 males and 13 females, with an average age of 52.22 years and an age of 42 to 66 years.

### Isolation of bone marrow mesenchymal stem cells (BMMSCs)

The bone marrow aspirates were harvested from the femur of trauma OA patients undergoing surgical treatment, and were collected in a heparin anticoagulation tube under aseptic conditions. Then, the bone marrow aspirates were centrifuged at 1500 g for 20 minutes, the fat layer was discarded, and this step was repeated twice. The BMMSCs were isolated using earlier published protocols [21]. Briefly, a total of 5 mL of Ficoll separation solution was added to a new centrifuge tube, and then the diluted bone marrow solution was added to the upper layer of Ficoll separation solution along the tube wall. Then the solution was centrifuged at 2500 g for 20 minutes. The white blood cell layer was removed, resuspended and centrifuged again to collect the pellet containing BMSCs. After resuspension, the collected BMSCs were cultured in DMEM/F-12 medium (Invitrogen Corp., Carlsbad, CA, USA) containing 10% fetal bovine serum (FBS). After 72 hours, discard the culture medium and non-adherent cells, and then add DMEM/F-12 complete medium for further culture and passage.

### Isolation and identification of exosomes

After reaching 50–60% cell confluence, BMSCs were further cultured in MesenGro® hMSC medium (StemRD, Burlingame, CA, USA) at 37°C and 5% CO_2_ for 48 hours. The conditioned medium was collected and centrifuged at 300 g for 15 minutes at 4°C, and then centrifuged at 2500 g for 15 minutes to remove dead cells and cell debris. After centrifugation, the supernatant was filtered through a 0.22 μM filter (Merck-Millipore, Darmstadt, Germany). The filtered solution was then transferred to a 15 mL Amicon Ultra 15 centrifugal filter device (Merck-Millipore, Darmstadt, Germany) and centrifuged at 4000 g until the volume of the supernatant was concentrated to about 200 μL. The ultrafiltrate containing exosomes was placed on top of a 30% sucrose/D2O pad, and then transferred to a sterile UltraClear™ tube (Beckman Coulter, Brea, CA, USA). The ultrafiltrate was then ultracentrifuged at 100,000 g (Beckman Optima XPN-100,Beckman, USA) for 1 hour at 4°C. The pellet was resuspended in 15 mL PBS and centrifuged again at 4000 g to concentrate the volume to about 200 μL. Observe the morphology of exosomes through a transmission electron microscope. Place the exosomes on a copper grid coated with formvar (StructureProbe, Inc., PA, USA). The grid was dried with 2% uranyl acetate, and then observed using a PhilipsMorgagni268D microscope (Philips, Amsterdam, Netherlands). The above process was repeated 3 times.

### Cell transfection

In order to explore the functions of miR-125a-5p, mimics, inhibitors and their negative controls were purchased from Ribbio (Guangzhou, China). The oligonucleotide transfection was performed using riboFECT™ CP reagent (RiboBioCo., Ltd., Guangzhou, China). In order to construct a lentiviral vector containing E2F2 overexpression, oligonucleotides were synthesized before the purified PCR product was co-cleaved with Xhol and MluI (PWPXL vector) and ligated with T4 ligase to construct a gene overexpression vector. The constructed core plasmid (16 μg) and two envelope plasmids PSPAX2 (12 μg) and PMD2G (4.8 μg) were co-transfected into HEK293T cells on a 6-well plate. The supernatant was collected 48 hours after transfection and concentrated with CentriconPlus-70 filter (UFC910096, Millipore). The experiment used lentivirus with a titer adjusted to 108TU/mL.

### Collection, isolation and culture of primary chondrocytes

Cartilage was excised from the subchondral bone, and then separated with 4 mg/mL protease and 0.25 mg/mL collagenase P. Cells were cultured in DMEM/F-12 (Gibco, Thermo Fisher Scientific) medium containing 5% FBS (Gibco, Thermo Fisher, Waltham, MA, USA) and 1% penicillin and streptomycin (Gibco, Thermo Fisher, Waltham, MA,USA). Chondrocytes are used for experiments within 3–7 days.

### Chondrocyte endocytosis

BMSCs were isolated with trypsin-EDTA and resuspended in 1 mL MesenGro® hMSC medium. To confirm whether the chondrocytes could take up exosomes derived from BMSCs, BMSCs were labeled using a green fluorescent dye Vybrant Dio (Molecular Probes, Carlsbad, CA, USA) before harvesting the exosomes [22]. After adding 5 μL Vybrant Dio solution to the suspension, the mixture was incubated at 37°C in 5% humidified CO_2_ for 15 minutes. The cells are then cultured in complete medium andseeded in a 35-mm glass-bottomed dish (D35-20-1-N, Cellvis, Mountain View, CA, USA) until the cell confluence reaches about 50–60%. The exosomes were then separated and cultured with chondrocytes for 6 hours. After washing 3 times with PBS to remove free exosomes, the cells were fixed with 4% paraformaldehyde for 15 minutes and stained with 4,6-diamino-2-phenylindole (DAPI) for 5 minutes. Finally, use the led fluorescence microscope (Mingmei, Guangzhou, China) to take images.

### RNA isolation and quantification

The total exosomal RNA isolation kit (Invitrogen, Carlsbad, CA, USA) was used to extract RNA from exosomes. The extracted RNA is then used for RT-qPCR detection. TRIzol reagent (Invitrogen, Carlsbad, CA, USA) was used to extract total RNA from chondrocytes or synovial MSC. For miRNA, human miRRT-qPCR detection kit (BioTNT, Shanghai, China) was used to synthesize cDNA, and U6 was used as an internal reference for miRNA. For mRNA, transscript® All-in-One-First-Strand cDNA synthesis supermix for RT-qPCR was used for cDNA synthesis. RT-qPCR was performed using TransStart®TopGreenRT-qPCRSuperMix (TransgenBiotech, Beijing, China), and β-actin was used as an internal reference for mRNA.

### Western blot

Western blot was used to separate total protein from cells using RIPA (Beyotime, Shanghai, China). The protein samples were electrophoresed on a 10% SDS-PAGE gel and electrotransferred to PVDF. The membrane was then blocked in Tris buffered saline containing 0.1% Tween20 and 5% skimmed milk powder Tween20 (TBST), and then incubated with primary and secondary antibodies. The primary antibodies are as follows: rabbit anti-CD63 (1:1000, ab134045), rabbit anti-CD9 (1:2000, ab92726), rabbit anti-CD81 (1:1000, ab109201), rabbit anti-Alix (1:1000, ab186429), rabbit anti Tsg101 (1:1000, ab30871), rabbit anti-MMP-13 (1:3000, ab39012), rabbit anti-collagen II (1:5000, ab34712), mouse anti-Aggrecan: 100, ab3778), rabbit anti-Sox9 (1: 1000, ab185230), rabbit anti-E2F2 (1:1000, ab235837) and rabbit anti-β-actin (1:1000, ab179467). The secondary antibodies are HRP-labeled rabbit anti-mouse immunoglobulin G (IgG) (1:5000, ab6728) and HRP-labeled goat anti-rabbit IgG (1:5000, ab6721). GAPDH was used as an internal reference for western blotting. Finally, use the ECL kit (Beyotime, Shanghai, China) to observe the staining, and use the BIORAD gel imaging system to measure the bands. All antibodies were purchased from Abcam Inc. (Cambridge, MA, USA).

### Transwell measurement of chondrocyte migration

After the chondrocytes were separated, 5 × 104 cells were seeded in the top chamber of a 24-well 8 μm multi-well plate. A total of 600 μL of complete chondrocyte culture medium containing exosomes was added to the basal compartment of the Transwell plate and incubated at 37°C for 12 hours. Then the upper chamber was fixed with 4% PFA for 15 minutes, and stained with 0.5% crystal violet for 10 minutes. Wipe the surface of the upper chamber to remove cells that have not migrated to the surface of the basolateral chamber. Finally, a Leica microscope (Leica Microsystems, Wetzlar, Germany) was used to take photos and statistical analysis of five randomly selected areas.

### Dual luciferase reporter detection

The wild-type (WT) and mutant (MUT) E2F2 plasmids were constructed to explore the targeting relationship between miR-125a-5p and E2F2. The recombinant plasmid was verified by restriction enzyme digestion and DNA sequencing. Add HEK293T cells to a 12-well plate. After 24 hours, cells were transfected with mimics miR-125a-5p and E2F2WT or E2F2MUT with Lipofectamine2000 reagent. The samples were then co-transfected with the pRL-TK plasmid expressing Renilla luciferase. 48 hours after transfection, the cells were lysed. The dual luciferase reporter gene detection system (Promega, USA) was used to detect firefly and Renilla luciferase activity.

### Traumatic OA mice model

48 healthy male C57BL/6 mice, aged 5–8 weeks, were randomly divided into 4 experimental groups: normal, traumatictraumatic OA, traumatictraumatic OA+exosomes and traumatictraumatic OA+ExosmiR −125a-5p. Each group consisted of 12 mice, and two mice were housed in each cage. After the mice were anesthetized with isoflurane, the right ankle and knee joints of the mice were placed in a custom processing table in a mechanical testing device. Applying a single mechanical load (1 mM/s to 12 N) to the ankle joint causes the tibia to move forward relative to the femur and extend the anterior cruciate ligament beyond the point of failure. Immediately after the injury, the joint is injected with 100 μL of 1011 particles/mL exocrine Then, let it recover from anesthesia, and then return to its cage. One hour after the injury, the mice were euthanized by inhalation of carbon dioxide and the joints were harvested [23]. All surface soft tissues were removed to separate the cartilage tissue of the joints.

### Statistical analysis

Our data analyses were accomplished with SPSS21.0 software (IBM, Armonk, NY, USA). Our data was shown as mean ± standard deviation. Independent samples t test was for the analysis on the comparison between two groups of unpaired data with normal distribution and homogeneity of variance. The comparison between multiple groups was analyzed with the measure of one-way variance (ANOVA) analysis and Tukey’s post-hoc test. Any value with p < 0.05 was statistically significant.

## Results

In this study, we explored the biological role and molecular mechanism of miR-125a-5p in OA. Our data showed that miR-125a-5p was lower expressed in traumatic OA cartilage tissues. miR-125a-5 could promote chondrocyte migration and alleviated cartilage degeneration caused by OA by targeting E2F2. In conclusion, our study showed the function of miR-125a-5p in traumatic OA via the E2F2, which meant that targeting these molecules may be a new method for the treatment of traumatic OA.

### E2F2 is a target of miR-125a-5p

**Figure 1.**
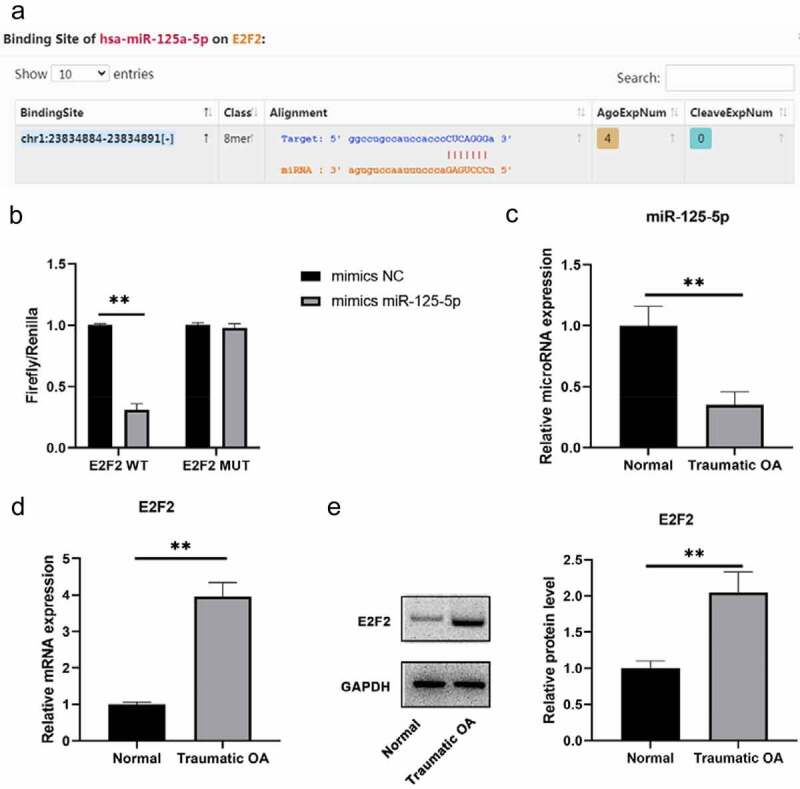
Distinct expression patterns of E2F2 and mir-125a-5p were observed in traumatic OA cartilage. A, Possible targeting relationship within mir-125a-5p and E2F2 was given by targetscan and Starbase databases. B, Verification of dual luciferase assay on the targeting relationship within mir-125a-5p and E2F2. C, Determination with RT qPCR assay on the expression of mir-125a-5p and E2F2 in normal cartilage and traumatic OA cartilage. D, Western blot was conducted to determinate the E2F2 expression in normal cartilage and traumatic OA cartilage. ** means P < 0.01, compared with normal group or mimics NC transfected cells

Low expression of miR-125a-5p is detected in traumatic OA cartilage tissues. We run a screening on the miRNAs over E2F2 upstream with the platforms of TargetScan and starBase databases (Figure 1(a)). After that, we carried out a dual luciferase reporter gene detection to verify the correlation within miR-125a-5p and E2F2 in cartilage tissue. Then it turned out that the transfection of miR-125a-5p impaired the luciferase activity by the way of binding to wild-type E2F2 3′UTR, which means transcription of E2F2 would also decrease; but the the luciferase activity with MUT E2F2 3′UTR sequence showed no change (Figure 1(b)). Subsequently, we obtained normal cartilage and traumatic OA cartilage specimens and made assays of RT-qPCR and western blot on miR-125a-5p and E2F2 expressions for our further explorations. High-expressed E2F2 and low-expressed miR-125a-5p were distinctly differential in traumatic OA cartilage, which indicated that their negative correlation (Figures 1(c-e)). Our findings suggested the targeting relation within miR-125a-5p and E2F2; morever, miR-125a-5p is low expressed in traumatic OA cartilage tissue.

### miR-125a-5p expression is detectable in bone marrow mesenchymal stem cells and their secreted exosomes

**Figure 2.**
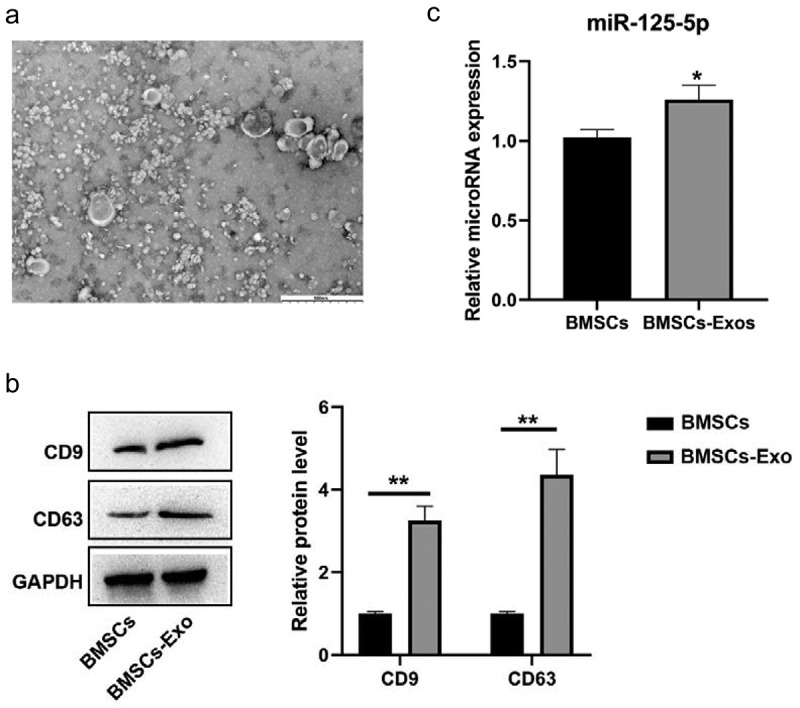
Identification of BMSCs and exosomes. A, Observation with transmission electron microscope on morphology of exosomes. B, Western blot analysis of secrete surface markers (CD63, CD9). C, RT-qPCR on the mir-125a-5p expressions in BMSCs and their exosomes. * P < 0.05, ** P < 0.01

We isolated exosomes from the medium with bone marrow mesenchymal stem cells growing. Our assays of transmission electron microscopy and western blot were for the identification on the exosomes secreted by BMSCs. Then it turned out that most of the vesicles were between 50–150 nm in size and presented a disc shape (Figure 2(a)). We made a further confirmation with western blot that exosomal markers CD63 and CD9 were highly expressed in our findings (Figure 2(b)). All the outcomes in this section indicated a successful exosomes isolation. Then following RT-qPCR assay was made for the determination on the miR-125a-5p expressions in both cells and exosomes, which affirmed the existence of miR-125a-5p in BMSCs and their secreted exosomes (Figure 2(c)).

### miR-125a-5p promotes the progression of chondrocytes and the secretion of ECM through E2F2

**Figure 3.**
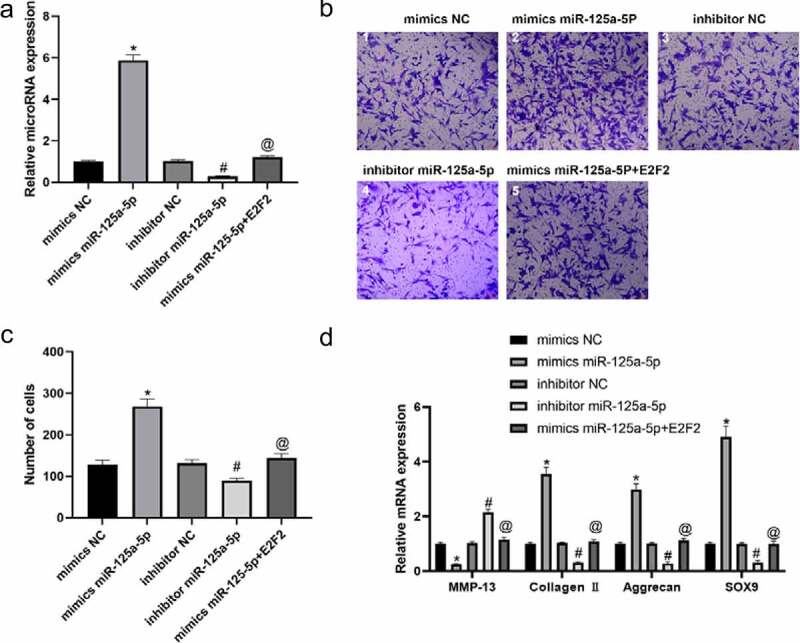
Targeting effect of mir-125a-5p to E2F2 leads to enhancement in both chondrocyte migration and ECM synthetic proteins. A, RT-qPCR assay on the mir-125a-5p expression in transfected chondrocytes. B and C, Migration analyses on chondrocytes. Scar bar = 100 μm. 1, mimics NC; 2, mimics miR-125a-5p; 3, inhibitor NC; 4, inhibitor miR-125a-5p; mimics miR-125a-5p + E2F2. D, RT-qPCR assays on the MMP-13, collagen II, aggrecan and Sox9 mRNA expressions. *Compared with the cells transfected with mimics NC, P < 0.05; # Compared with the cells transfected with inhibitor NC, P < 0.05; @ Compared with cells transfected with mimics mir-125a-5p, P < 0.05

**Figure 4.**
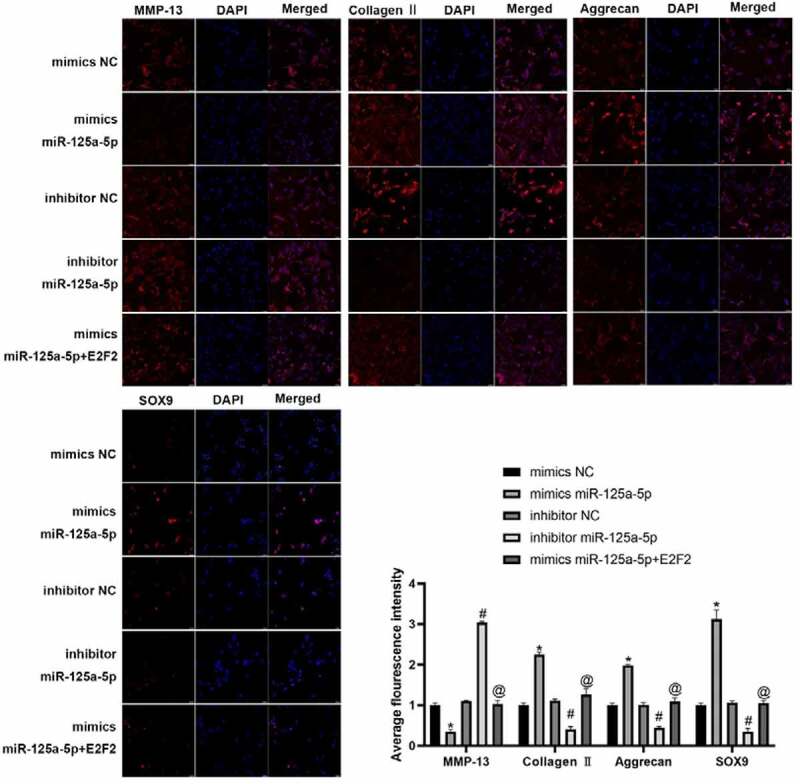
The protein expression of MMP-13, collagen II, aggrecan and Sox9 in chondrocytes were detected by immunofluorescence. * Compared with the cells transfected with mimics NC, P < 0.05; # Compared with the cells transfected with inhibitor NC, P < 0.05; @ Compared with cells transfected with mimics mir-125a-5p, P < 0.05

We isolated the chondrocytes from clinical normal cartilage tissue and con-transfected them with miR-125a-5p and E2F2 (Figure 3(a)). Our Transwell experiment was performed for the assessment on cellular migration ability, whose results showed that the cells with mimics miR-125a-5p transfection manifested an enhancement in cellular migration in contrast to the ones with mimics NC transfected; the cells with miR-125a-5p inhibitor transfection showed an impaired migration ability versus the inhibitor NC group. In contrast with the ones with miR-125a-5p mimics transfection, migration ability of the cells with both miR-125a-5p and E2F2 con-transfected was notably suppressed (Figures 3(b,c)). RT-qPCR and immunofluorescence results showed that the Collagen II, aggrecan and SOX9 contents in the chondrocytes with miR-125a-5p mimics transfected were significantly increased, while the level of MMP-13 was lower than the mimics-NC transfected cells. We also found that the content of Collagen II, aggrecan and SOX9 in chondrocytes turned down after miR-125a-5p inhibitor transfection; reversely, expression of MMP-13 up-regulated. Besides that, the Collagen II, aggrecan and SOX9 contents in the cells with miR-125a-5p transfection became down-regulated. However, when compared with the one of miR-125a-5p mimics transfection, the levels of E2F2 and MMP-13 in the miR-125a-5p inhibitor transfection were higher (Figures 3(d) and 4). In summary, the above results indicate that miR-125a-5p promotes the E2F2 migration to chondrocytes as well as the secretion of ECM.

### Exosomal miR-125a-5p accelerates chondrocyte migration and ECM secretion

**Figure 5.**
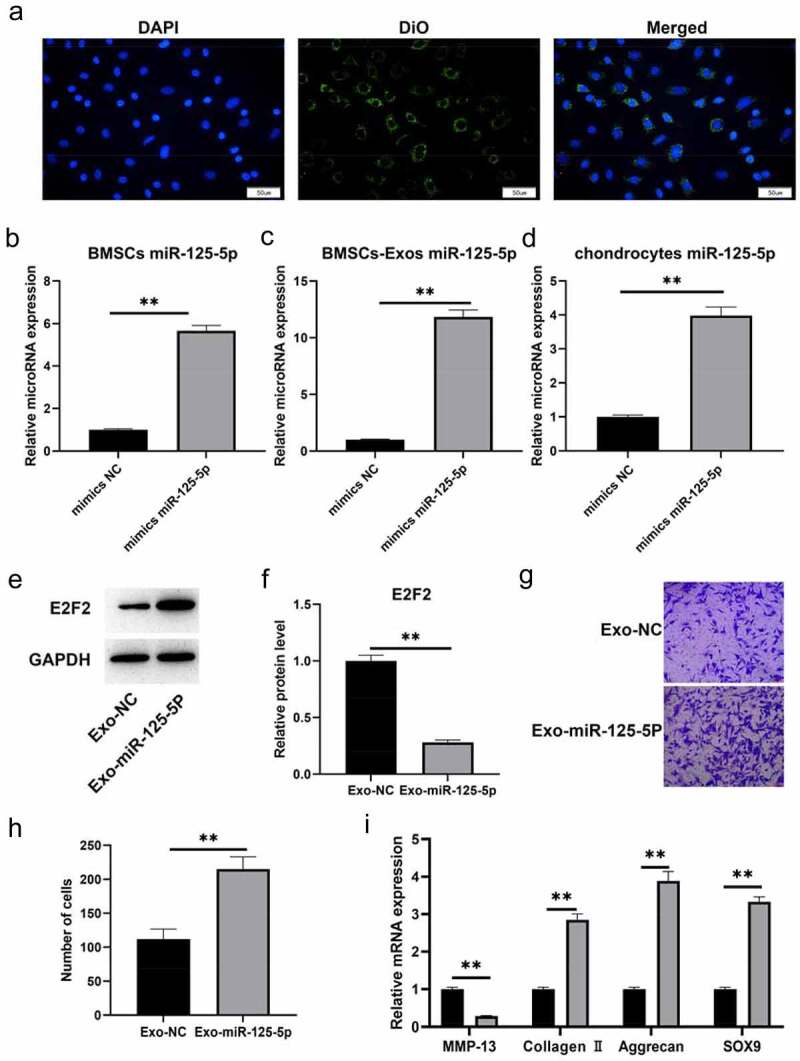
Exosomal miR-125a-5p accelerates chondrocyte migration and secretion of ECM synthetic proteins. A, The typical immunofluorescence pattern of DIO (green)-labeled BMSCs-Exosabsorbed by chondrocytes was that their nuclei were stained with DAPI (blue). B, The RT-qPCR assay to determinate the mir-125a-5p expression in transfected BMSCs. C, The RT-qPCR determination on the mir-125a-5p expression in the exosomes of transfected BMSCs. D, The RT qPCR determination on the mir-125a-5p expression in chondrocytes. E and F, Western blot analysis on the E2F2 protein in exosome-treated chondrocytes. G and H, Migration analysis on chondrocytes. I, The RT-qPCR assay on the mRNA determination of MMP-13, collagen II, aggrecan and Sox9 expressions. ** P < 0.01, represents a comparison with the exoago NC or cellago NC group

Before obtaining exosomes, we labeled BMMSCswith a green fluorescent lipophilic dye (Vybrant-DiO) so that the exocrine of bone marrow mesenchymal stem cells could be detected. With the help of those assays, we profiled the roles of miR-125a-5p and the absorbing effect of chondrocytes to exosomes. We had the chondrocytes incubated for 6 hours with the exosomes of labeled cells, then we found the DIO-labeled exosomes around the perinuclear area of chondrocytes (Figure 5(a)), which indicated the endocytosis of chondrocytes to exosomes. Subsequently, we conducted a miR-125a-5p over-expression in bone marrow mesenchymal stem cells (Figure 5(b)), then we isolated their exosomes for following experiments. RT-qPCR outcome (Figure 5(c)) revealed that the miR-125a-5p content in bone marrow mesenchymal stem cells secretory exosomes was increased after exogenous miR-125a-5p over-expression. In addition, the miR-125a-5p expression in chondrocytes was subjected to a further test after endocytosis. Results of Figures 5(d-f) showed that the miR-125a-5p expression in chondrocytes become up-regulated after endocytosis, whereas the E2F2 turned down. After that, we gave a treatment of the exosomes isolated above to chondrocytes, and determined their migration ability with Transwell assay. Then the outcomes turned out that the migrating cell number of the one with EXO miR-125a-5p -ago transfection increased notably higher than the cells with EXOago-NC transfection (Figures 5(g,h)). Additionally, the RT-qPCR outcome indicated that the collagen II, aggrecan and SOX9 expressions in the chondrocytes with EXOmiR-125a-5p transfection was higher than the ones with EXOago-NC, but the MMP-13 expression in the EXOmiR-125a-5p decreased (Figure 5(i)).

### Exomir-125a-5p inhibits cartilage degradation

**Figure 6.**
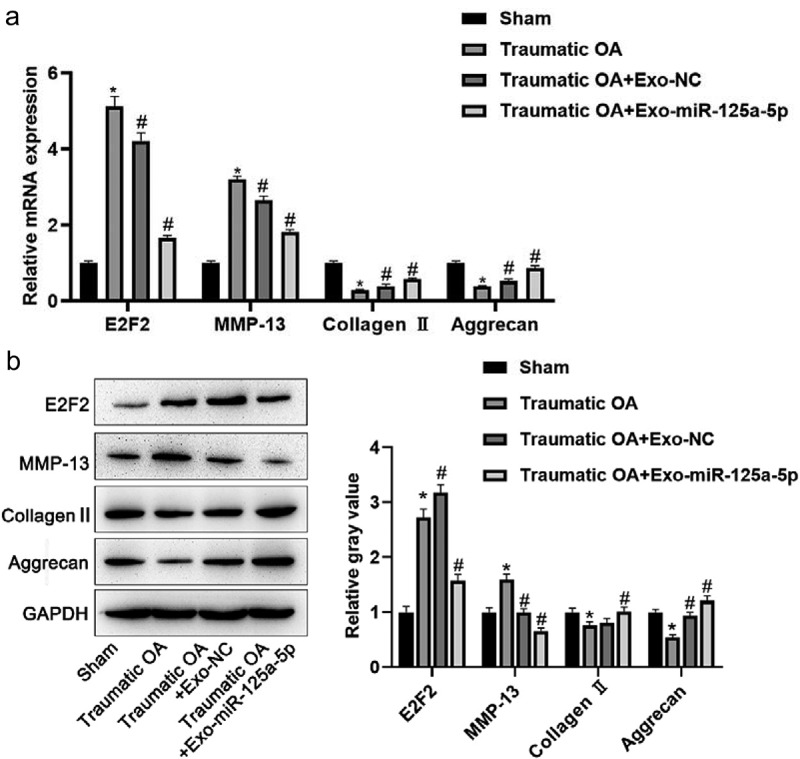
Exo mir-125a-5p inhibits cartilage degradation. A, RT-qPCR assays for the determination to E2F2, collagen II, aggrecan and MMP-13 expressions. B, Protein expressions of E2F2, collagen II, aggrecan and MMP-13 were detected by western blot.*P < 0.05, compared with sham operated mice; #P < 0.05, compared with traumatic OA group

In this section, we had a further study to the exosomal mir-125a-5p function in OA with the mice model of traumatic OA, and we used this model to run a test on exosomal mir-125a-5p preventive potential in traumatic OA. Mice in OA group showed a decreasing level of cartilage matrix after trauma; we then made a comparison with sham operated mice, and it turned out that the collagen II and aggrecan expressions in damaged cartilage was down-expressed, while those of E2F2 and MMP-13 became elevated. Losses of cartilage matrix in OA mice with exo injections were significantly less when compared with the OA mice. The exomir-125a-5p injection into OA mice was not capable of ceasing cartilage matrix losing, but the severity saw lower than the OA mice with the exo injection. Collagen II and aggrecan expressions were slightly inferior versus the normal group, but higher than the traumatictraumatic OA mice as well as the traumatictraumatic OA mice with the exo injection (Figure 6). All these findings proved the inhibitory effect of mir-125a-5p to OA in its early stage after trauma, and we could also infer that mir-125a-5p is able to slow down further injury in knee cartilage.

## Discussion

OA is a prevalent joint disease of inflammation often happening to elderly population [24]. There are about 240 million people worldwide suffering from OA, whose main cause is commonly defined as the cumulative tissue damage in the elder because of mechanical erosion and aging [25]. Currently, there is no effective OA diagnosis at its early stage [25]. Furthermore, conventional treatment to OA often comes along with side effects as well as the surgery with invasiveness [3]. We presented an evidence in this study that the exosomal mir-125a-5p generated by MSCs targets E2F2 to inhibit OA chondrocytes degeneration. We found a negative correlation within E2F2 and mir-125a-5p in traumatic OA cartilage that the E2F2 gene was highly expressed when the content of mir-125a-5p was low. E2F2 is a broad player in many diseases as well as their progression. For example in osteoarthritis, high level of E2F2 mRNA was detected in the cartilages of the patients with OA [26]. Besides, imblanced expressions of E2F2 in osteoarthritis suggests a deterioration in cartilage catabolism and abnormal anabolism of damaged cartilage [26]. E2FE is also a participant in the regulation of cellular inflammatory mediators [27]. In this study, we revealed a fact that gene E2F2 was found over-expressed in both traumatic OA patients’ cartilage and our mice models. miRNA, another molecule of our study, which is a kind of endogenous non-coding RNA category with a post-transcriptional regulation to encoding genes [28]. miRNAs’ correlation with OA pathogenesis has been dug out recently [29]. It was reported that the mRNA expression of mir-125a-5p was down-expressed in plasma of patients with knee OA [30]. Targeting regulation of miR-125a-5p and ship1 was found to modulate inflammatory response [31]. It was also found that the transfection of mir-125a-5p mimics in vivo into an acidic environment would repress Th17-induced inflammation [32]. Further, mir-125a-5p is a regulator in the microglia mediated neuroinflammation of hypoxic-ischemic encephalopathy [33].

It is consistent as the findings above that we found the expression of mir-125a-5p is inhibited in traumatic OA cartilage. mir-125a-5p is also a powerful enhancer in both E2F2 chondrocyte migration and ECM secretion. miRNAs have been proved to be involved in the migration of chondrocytes and cartilage degradation [34–36]. Overexpressed miR-195 facilitates chondrocyte migration in a way of targeting GIT1 [37], whereas mir-486-5p over-expression inhibits chondrocyte migration by inhibiting Smad2 [38]. In addition, mir-139 has been reported to inhibit chondrocyte migration [39]. Herein, we verified the trait of mir-125a-5p promoting chondrocytes migration. On the contrary, E2F2 is the inhibitory factor in chondrocytes migration. Extracellular matrix (ECM) is a complex network composed of structural proteins and glycosaminoglycans [40], imbalance of ECM results in many diseases’ development [40]. As far as the importance of ECM, miR-125a-5p is an answer in maintaining its stability by inhibiting E2FE expression.

It was reported in previous studies that the articular cartilage degradation is one of the causes of OA development [41]. Chondrocyte hypertrophy is the key step as well as the final one in both OA pathogenesis and chondrocyte differentiation [42]. Low accumulated collagen inside ECM has been revealed a key issue in damaged cartilage tissue; furthermore, aggrecan plays a crucial role in ECM growth plate cartilage [43]. Regulator Sox9 is a key transcription factor in chondrocyte development [44], whereas MMP-13 is the another one in ECM component degradation [45]. On the basis of these findings, we could see that the expressions of collagen II, aggrecan and Sox9 are notably elevated after mir-125a-5p over-expression, whereas the expression of MMP13 turned greatly decreased. We could hence infer that over-expressed mir-125a-5p leads to an inhibition to cartilage degeneration. Exosomes, a kind of small membrane encapsulated vesicles, are the signal mediators within cells with their ability to carry miRNA and other bioactive substances from cells to cells. They are also the participants in chondrocyte migration [46]. For example, Synovial MSCs are able to prompt migration of articular chondrocytes with their mir-140-5p [47]. mir-125a-5p has a same ability in promoting proliferation and migration, whereas it accomplishes that by by targeting E2F2.

## Conclusion

In this study, we revealed a negative correlation within mir-125a-5p and E2F2 in traumatic osteoarthritis patients’ cartilage as well as in our mice model. Chondrocytes are given an ability to endocytose mir-125a-5p, so that endogenous expressions of E2F2 and MMP-1 would be suppressed, meanwhile the expressions of collagen II, aggrecan and Sox9 as well, which brings about a consequence with chondrocyte migration promoted whereas cartilage degeneration repressed.

## Highlights

Exosomes miR-125a-5p reduced the degradation of chondrocyte extracellular matrix. Exosomes miR-125a-5p promoted chondrocyte migration.

Exosomes miR-125a-5p inhibited cartilage degeneration in vivo.

E2F2 is the target of exosomes miR-125a-5.
